# Intracellular Ionic Remodeling During Fetal Development of Hereditary Cardiomyopathy of the Hamster

**DOI:** 10.3390/pathophysiology33020037

**Published:** 2026-06-01

**Authors:** Ghassan Bkaily, Alexandre Normand, Ashley Jazzar, Houssein Najibeddine, Danielle Jacques

**Affiliations:** Department of Immunology and Cell Biology, Faculty of Medicine and Health Sciences, Université de Sherbrooke, Sherbrooke, QC J1H 5N4, Canadaashley.jazzar@usherbrooke.ca (A.J.); houssein.najibeddine@usherbrooke.ca (H.N.)

**Keywords:** cardiomyopathy, fetal cardiac development, NHE-1, sodium, calcium, free radicals

## Abstract

Background/objectives: The development of hereditary cardiomyopathy associated with early death was known to take place during the patients’ lifetime. Previous work showed that remodeling of heart cells in hereditary cardiomyopathy of hamsters (HCMH) took place as early as during postnatal development. The present work tested the hypothesis that cardiomyocyte remodeling in HCMH occurred early in fetal development. Methods and results: Using quantitative 3D confocal microscopy associated with ionic and immunofluorescence, our results showed that, as in the adult heart, cardiomyocytes isolated from 15-day-old fetal HCMH hearts showed an increase in intracellular calcium, sodium, ROS, and sodium-hydrogen exchanger 1 (NHE-1) when compared to age-matched cardiomyocytes of a normal hamster (NH). Conclusions: These results demonstrated that hereditary cardiomyopathy occurred during fetal heart development, and that early treatment with an NHE-1 blocker might have prevented the development of hereditary cardiomyopathy and heart failure that occur during the hamsters’ lifetime.

## 1. Introduction

Genetic heart diseases were commonly classified into three categories: (1) structural, such as hypertrophic, dilated, and arrhythmogenic of the right ventricular; (2) congenital cardiomyopathy, such as tetralogy of Fallot; and (3) channelopathic, such as long QT syndrome [1,2] and Brugada syndrome [3,4,5]. In addition, our group showed that in animal models of the cardiomyopathic hamster and in skeletal muscle from patients with human Duchenne muscular dystrophy, the early fetal slow sodium channel was continuously expressed [6,7]. Congenital heart diseases are structural abnormalities affecting the heart and/or vessels, and were linked to increased morbidity throughout life and reduced survival [4,8,9]. They arose during embryogenesis and involved abnormal cardiac morphogenesis, including atrial septal defect, ventricular septal defect, and cardiac outflow tract malformations [10,11]. Morphological genetic heart diseases, such as cardiomyopathies and inherited arrhythmia syndromes, were conditions in which myocardial structure may initially appeared intact, yet cardiac contractile performance or electrical activity was compromised [12,13,14,15]. At the molecular level, many genetic heart diseases were caused by mutations in sarcomeric [16,17,18], cytoskeletal [19,20,21], ion-channel [2,5,6], or membrane-associated proteins [22], which disrupted excitation–contraction coupling and altered intracellular signaling pathways [23,24,25].

Calcium, a key regulator of cardiac contraction, plays an essential role in excitation–contraction coupling [26]. Thus, impaired calcium handling led to various heart dysfunctions, including catecholaminergic polymorphic ventricular tachycardia—which is mainly caused by uncontrolled calcium release from the sarcoplasmic reticulum [27]—and hypertrophic cardiomyopathy, often linked to mutations in sarcomeric genes encoding contractile proteins [28,29].

Cardiac sodium channels are essential for proper heart function; therefore, dysfunction in these channels can lead to life-threatening arrhythmias [30]. Such dysfunction occurs in myocardial ischemia or heart failure, as well as in inherited disorders caused by mutations in the SCN5A gene, which encodes the Nav1.5 cardiac sodium channel [30]. This mutation resulted in increased sodium influx, prolonged depolarization, and, consequently, increased calcium influx through the sodium–calcium exchanger [30].

Disruptions in ionic homeostasis during cardiac development had important consequences as fetal cardiomyocytes underwent rapid maturation of ion channels and exchangers during gestation [31]. Therefore, perturbations in ionic homeostasis during fetal life can potentially influence cardiomyocyte structure and function, thereby predisposing the heart to disease later in life [31,32].

The objective of the present work was to verify whether ionic remodeling and the increase in the relative density of NHE1 and ROS level during the postnatal development of HCMH took place before birth during fetal development.

## 2. Materials and Methods

### 2.1. Isolation and Culturing of Cardiomyocytes

For in vitro isolated-cell studies, as previously cited, we will determine the semi-quantitative levels (per µm^3^) of intracellular Na^+^, Ca^2+^, and ROS, as well as the relative fluorescence density of NHE1. This will be done in single intact cardiomyocytes freshly isolated from the ventricles of a 15-day-old fetal normal hamster (NH) (Charles River) and an aged-matched fetal hereditary cardiomyopathic hamster (HCMH) (our UMX-7.1 colony) using a standard technique currently used in our laboratory [6,33,34]. The isolation method is similar to that previously described [33]. In brief, the pregnant normal hamster and cardiomyopathic hamster of the UMX-7.1 line [35] are open, and the fetal hamsters are collected at the age of 15 days. Their hearts are dissected, washed, and placed in M199 medium (Gibco-BRL, Burlington, ON, Canada) containing antibiotics and 0.1% (50 U/mL) collagenase for 15 min. Next, the medium is replaced with a fresh, collagenase-free M199 solution containing antibiotics. The ventricular cardiomyocytes are then gently scraped with a sterile scalpel blade, and the cell-containing medium is collected and centrifuged using the same conditions as those described previously [36]. Cells were cultured in M199 medium containing 10% fetal bovine serum and 50 IU/mL penicillin-G-potassium (Ayerst, Toronto, ON, Canada) to allow cardiomyocytes to attach to the bottom of the culture dish and were used within 2–3 h after isolation. This technique guarantees the purity of cardiomyocytes. These isolated cells are used to determine whole-cell and nuclear volumes, intracellular Na^+^, Ca^2+^, and ROS, as well as NHE1 density, using quantitative 3D confocal imaging [34].

### 2.2. Quantitative 3D Confocal Imaging

For all aims, cells are studied using quantitative 3D laser confocal microscopy, and whole-cell and nuclear volume measurements are obtained using the Syto-11 fluorescent probe by digitally removing the nucleus from the surrounding cytoplasm [33,34]. Different fluorescent probes will be used (Fluo-4 for Ca^2+^, sodium green for Na^+^, and DCFH-DA for ROS) [34]. In brief, cardiomyocytes loaded with the fluorescent probe or primary and secondary antibody-loaded (for NHE1 measurement) are examined with an MRC1024 Krypton/Argon and UV laser (BioRAD, Mississauga, ON, Canada) confocal system. Pinhole size, image size, pixel size, and step size are set to 50 µm, 512 × 512 pixels, 0.34 µm, and 0.5 µm, respectively. In all experiments, laser line intensity, photometric gain, PMT settings, and filter attenuation are kept rigorously constant throughout the experimental procedures [34]. The recorded serial *Z*-axis optical scans are analyzed by a Silicon Graphics O2 analysis station equipped with Molecular Dynamics Imagespace 3.2 analysis and Volume Workbench software modules [33,34]. The latter corrects for any axial and spherical aberrations that may occur in image acquisition [33,34]. In brief, as described previously for single-cell studies [33,34], the intensity of fluorescence is measured throughout the entire volume of each compartment (cytosol + nucleus or nucleus including the nuclear membranes) per µm^3^. Whole volume quantitative 3D eliminates any possible problem concerning the drifting of the Z-line, permitting us to measure and normalize (per µm^3^) fluorescence intensity accurately [33,34]. This method also permits us to determine the localization and distribution in each compartment [33,34]. Isolated cells from at least five pregnant females will be used for each experimental series. Indirect immunofluorescence: Indirect immunofluorescence of cells is performed as previously described [33]. Briefly, after fixation, permeabilization, and overnight incubation with the corresponding primary antibody, the samples are incubated with the secondary antibodies (1 µg/mL) coupled to the fluorescent probe Alexa-Fluor (Molecular Probes, Eugene, OR, USA). For NHE1 immunofluorescence labeling, the freshly isolated cells will be immediately used for immunostaining using a commercially available NHE1 monoclonal antibody that was used in our experiments and found to be highly specific for NHE1. The Biorad Krypton-Argon and UV-laser confocal scanning microscope system will be used. Measurements will be expressed per µm^3^ using ImageSpace 3.2 in the whole heart using a double-blind procedure [37].

### 2.3. Statistics

Fluorescence intensity values are expressed as means ± SEM. N is the number of pregnant females (3–5), and n is the number of cells from at least 3–5 different experiments. Statistical significance was determined using a repeated-measures ANOVA for matched values, followed by a Bonferroni multiple-comparison test to assess the significance of the results. *p* values of less than 0.05 were considered significant.

## 3. Results

### 3.1. Difference Between the Resting Whole-Cell, Cytosolic, and Nuclear Calcium Levels in Isolated Cardiomyocytes of 15-Day-Old Fetal NH and HCMH

Using quantitative 3D confocal microscopy, the objective of this series of experiments was to determine whether there are differences in calcium resting levels between isolated cardiomyocytes from 15-day-old fetal NH and HCMH.

**Figure 1:**
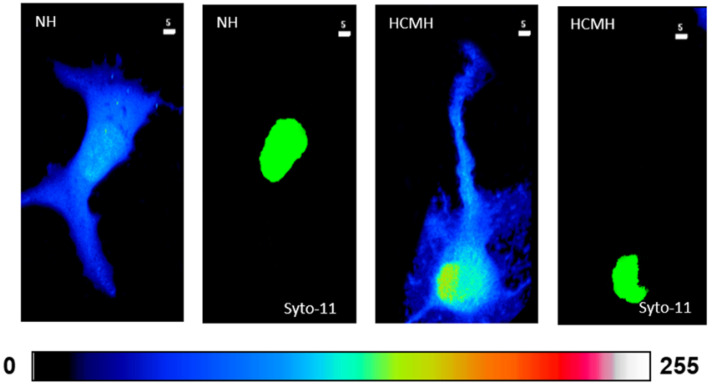
Examples of a typical Ca^2+^ fluorescence intensity of top view image quantitative 3D top-view image of cardiomyocytes from normal from 15-day-old fetal normal hamster (NH) and HCMH. The pseudo color scale represents NHE1 fluorescence intensity ranging from 0 (no fluorescence, black color) to 255 (maximum fluorescence, white color). The white scale bar is μm. The green color of the nucleus (Syto11) has no measurable meaning.

**Figure 2:**
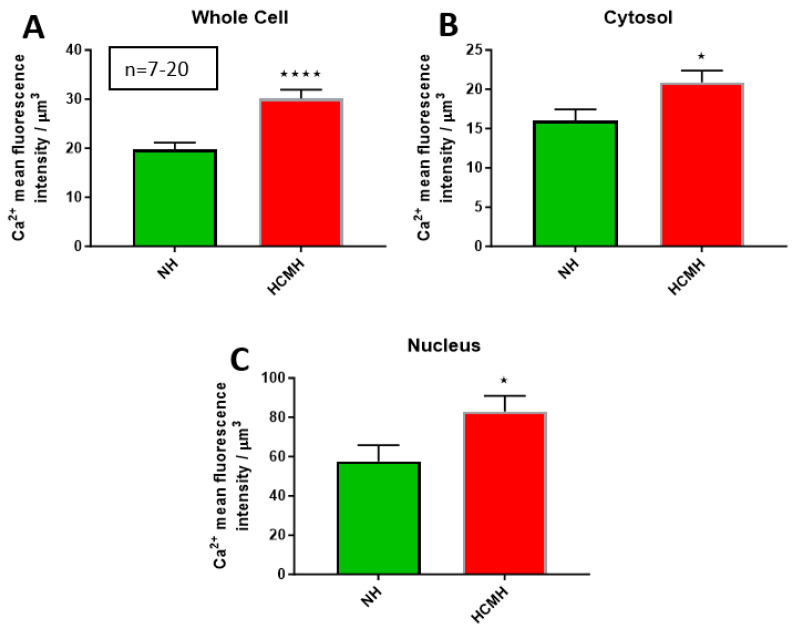
Resting whole-cell, cytosolic, and nuclear calcium levels in cardiomyocytes of 15 days old of fetal NH and HCMHs. Histograms showing the whole-cell (**A**), cytosolic (**B**), and nuclear (**C**) resting calcium levels of 15-day-old fetal cardiomyocytes of NH and HCMH. The values are represented as mean ± standard error of the mean. * *p* < 0.05, and **** *p* < 0.0001. Calcium’s relative concentration is expressed in μm^3^.

In the first series of experiments, after 24 h in culture, once the cells were loaded with the calcium fluorescence probe Fluo-4, we recorded calcium fluorescence levels in the whole cell, cytosol, and nucleus. Figure 1 shows a typical example and Figure 2A–C summarizes the results, respectively, in the whole cell, cytosol, and nucleus. As shown in this figure, there was a significantly higher level of calcium in the whole cell (*p* < 0.0001), cytosol (*p* < 0.05), and nucleus (*p* < 0.05) in the cardiomyocytes from 15-day-old fetal HCMH when compared to the level of intracellular calcium of 15-day-old fetal NH.

### 3.2. Difference Between the Resting Whole-Cell, Cytosolic, and Nuclear Sodium Levels in Isolated Cardiomyocytes of 15-Day-Old Fetal NH and HCMH

As in Section 3.1, using quantitative 3D confocal microscopy, the objective of this series of experiments was to determine whether there are differences in sodium resting levels between isolated cardiomyocytes from 15-day-old fetal NH and HCMH.

In this second series of experiments, after 24 h in culture, cells were loaded with the sodium fluorescence probe sodium green to determine the resting sodium levels in the whole cell, cytosol, and nucleus. As shown in Figure 2A–C, the resting sodium levels in the whole cell, cytosol, and nucleus were significantly (*p* < 0.05) different between NH cardiomyocytes when compared to those of age-matched fetal HCMH.

### 3.3. Difference Between the Resting Whole-Cell, Cytosolic, and Nuclear ROS Levels in Isolated Cardiomyocytes of 15-Day-Old Fetal NH and HCMH

As in Section 3.1, using quantitative 3D confocal microscopy, the objective of this series of experiments was to determine whether there are differences in ROS resting levels between isolated cardiomyocytes from 15-day-old fetal NH and HCMH.

**Figure 3:**
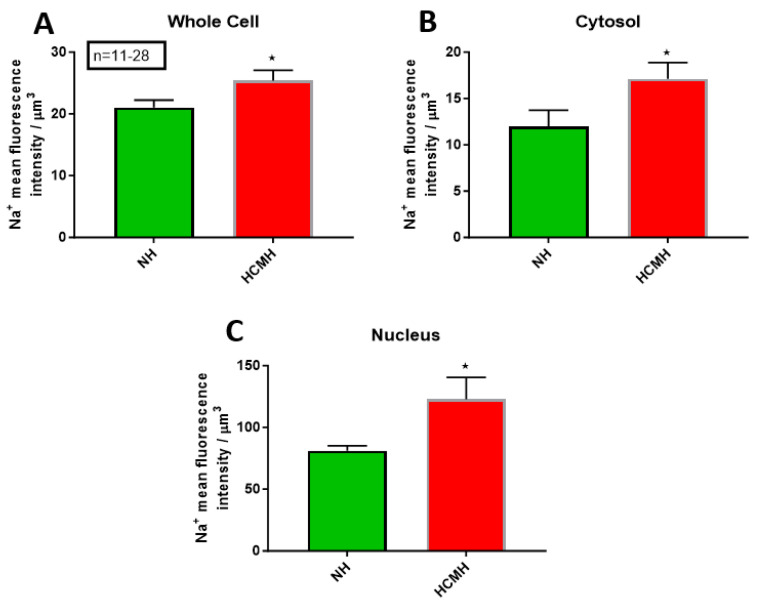
Resting whole-cell, cytosolic, and nuclear sodium levels in cardiomyocytes of 15 days old of fetal NH and HCMHs. Histograms showing the whole-cell (**A**), cytosolic (**B**), and nuclear (**C**) resting sodium levels of 15-day-old fetal cardiomyocytes of NH and HCMH. The values are represented as mean ± standard error of the mean. * *p* < 0.05. Sodium’s relative concentration is expressed in μm^3^.

**Figure 4:**
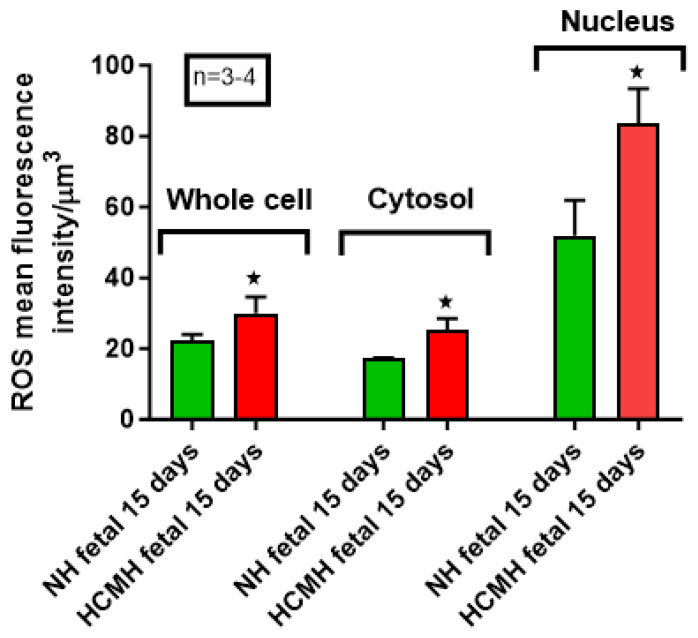
Resting whole-cell, cytosolic, and nuclear ROS levels in cardiomyocytes of 15 days old of fetal NH and HCMHs. Histograms showing differences in the resting levels of intracellular ROS between the whole-cell, cytosolic, and nuclear compartments of NH and HCMH cardiomyocytes. The values are represented as mean ± standard error of the mean. * *p* < 0.05. ROS’ relative concentration is expressed in μm^3^.

In this third series of experiments, after 24 h in culture, we loaded cells with the ROS fluorescence probe DCFH-DA to determine resting ROS levels in the whole cell, cytosol, and nucleus. As shown in Figure 3 and Figure 4, the resting ROS levels in the whole cell, cytosol, and nucleus were significantly (*p* < 0.05) different between NH cardiomyocytes when compared to those of age-matched fetal HCMH.

### 3.4. Difference Between the Relative Density of the Whole-Cell, Cytosolic, and Nuclear NHE1 Levels in Isolated Cardiomyocytes of 15-Day-Old Fetal NH and HCMH

As in Section 3.1, using quantitative 3D confocal microscopy, the objective of this series of experiments was to determine whether there are differences in the relative density of NHE1 between isolated cardiomyocytes from 15-day-old fetal NH and HCMH.

**Figure 5:**
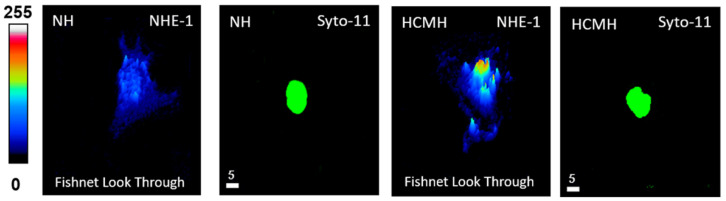
Examples of a typical NHE1 fluorescence intensity and distribution Fishnet Lookthrough quantitative 3D image of cardiomyocytes from normal from 15-day-old fetal normal hamster (NH) and HCMH. The pseudocolor scale represents NHE1 fluorescence intensity ranging from 0 (no fluorescence, black color) to 255 (maximum fluorescence, white color). The white scale bar is μm. The green color of the nucleus (Syto11) has no measurable meaning.

**Figure 6:**
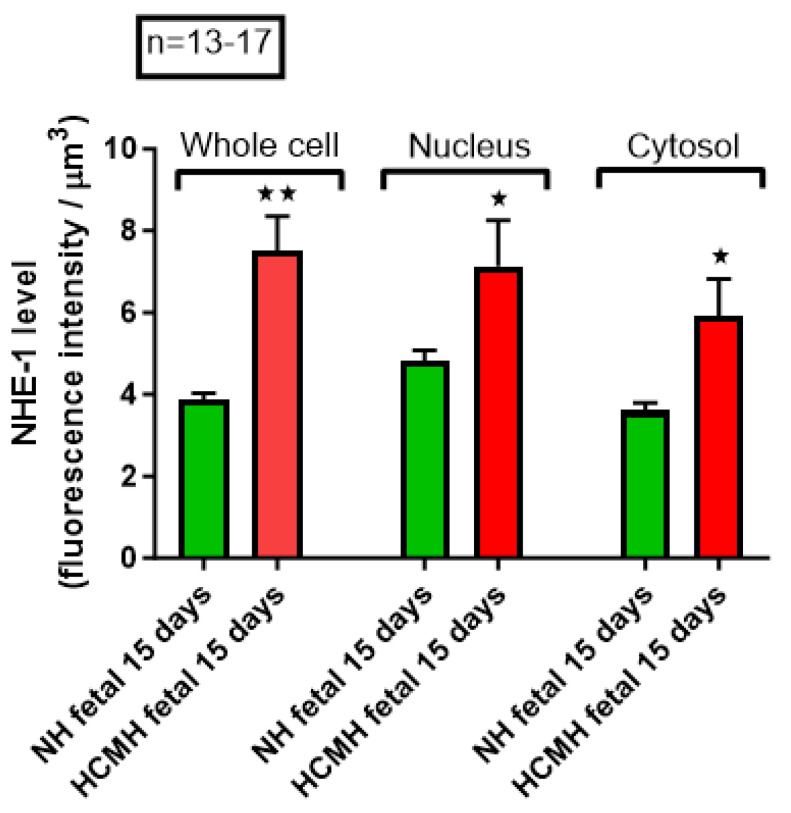
Relative density of NHE-1 in the whole cell, nucleus, and cytosol in cardiomyocytes of 15 days old of fetal NH and HCMHs. Histograms showing an increase in the relative density of NHE-1 level in the whole cell, nucleus, and cytosol of cardiomyocytes of HCMH. The values are represented as mean ± standard error of the mean. * *p* < 0.05, ** *p* < 0.01. NHE-1 relative density is expressed in μm^3^.

In this last series of experiments, after 24 h in culture, we used immunofluorescence with an NHE1 monoclonal antibody to determine the relative density of NHE1 in the whole cell, cytosol, and nuclei. Figure 5 showed a typical example and Figure 6 summarizes the results. As shown in Figure 6, the relative density in the whole cell, cytosol, and nucleus was significantly different (*p* < 0.01 and *p* < 0.05) between NH cardiomyocytes when compared to those of age-matched fetal HCMH.

## 4. Discussion

Intracellular calcium overload in ventricular cardiomyocytes has been reported in several cardiac diseases, including ischemia/reperfusion injury [38,39], necrosis [40], hypertrophy [26], and heart failure [37]. This abnormal disturbance in intracellular Ca^2+^ homeostasis has been attributed to dysfunctional direct and indirect Ca^2+^ transport systems, including Ca^2+^ channels (T-, L-, and R-types), exchangers (Na^+^/Ca^2+^ and Na^+^/H^+^), pumps (Ca^2+^-ATPase and Na^+^/K^+^-ATPase), as well as Ca^2+^ release channels such as ER/SR ryanodine and IP3 receptors [26,31,37,38,41,42,43,44,45]. In addition, Ca^2+^ overload in heart diseases has been reported to occur at both the mitochondrial and nuclear levels [26,37,39,46,47], depending on the extent of cytosolic Ca^2+^ overload [6,26,34,48].

Our research group clearly demonstrated this intracellular Ca^2+^ overload, occurring indirectly through the Na^+^/Ca^2+^ exchanger, and the increase in Na^+^ efflux in response to intracellular Na^+^ overload [26,37]. This may represent the mechanistic link between the abnormal increase in NHE-1 density/activity and intracellular Ca^2+^ overload during ventricular cardiomyocyte hypertrophy [26,37,49,50]. In our previous work on hereditary cardiomyopathy, we showed that cardiac cell necrosis is characterized not only by calcification but also by sodification, and that these alterations occur in the absence of measurable hypertrophy [26,37].

Abnormal remodeling of ventricular cardiomyocytes is generally associated with mitochondrial Ca^2+^ overload and enhanced mitochondrial activity, which promote the generation of reactive oxygen species (ROS) [51,52]. These ROS were further reported to contribute to intracellular Ca^2+^ accumulation [53,54]. The remodeling of ventricular cardiomyocytes following intracellular Ca^2+^ overload may result, at least in part, from the activation of Ca^2+^-binding proteins such as calmodulin, calcineurin, protein kinase C, and S100 proteins, as well as their downstream nuclear signaling pathways that modulate abnormal gene expression associated with hereditary cardiomyopathy and the early death of DMD/BMD patients.

Most of the work, if not all, on hereditary cardiac disease has been conducted during the lifetimes of human patients [55], and animal models such as HCMH [37]. These studies in HCMH clearly showed that, in its early stage, hereditary cardiomyopathy was associated with cardiomyocyte remodeling of ionic homeostasis [33,37], including calcium and sodium, without morphological changes [37]. Our group reported that morphological changes in HCMH were associated with increased density and activity of the NHE-1, and that this was prevented by treatment with the NHE-1 inhibitor, rimeporide [37]. Our group showed that remodeling of ionic homeostasis in HCMH took place as early as the newborn stage [6,34,37].

The objective of the present work was to verify whether ionic remodeling and the increase in the relative density of NHE1 and ROS during the postnatal development of HCMH [33,37] took place before birth during fetal development. Our findings showed that ionic remodeling as well as NHE1 relative density took place during fetal development in HCMH. These results suggest that development of treatment of hereditary cardiomyopathy should not only have targeted postnatal development of the disease [33,37] but also cardiomyocyte remodeling during in utero development.

In addition, the present work showed that the fetal ionic remodeling of heart cells in HCMH, as in the adult situation, is associated with increases in the density of NHE-1 and ROS levels [35,37,56]. The increase in ROS levels during fetal remodeling of cardiomyocytes in HCMH could be due, as in adult HCMH [37], to increased cellular metabolic activity and mitochondrial dysfunction caused by cytosolic and mitochondrial calcium overload [57,58]. The increase in intracellular sodium during fetal heart cell development of HCMH could be due directly or indirectly to factors such as: (1) the increase in sodium influx through the early fetal slow sodium channel [6]; (2) the increase in Ca^2+^ influx via increase in window T-type Ca^2+^ current [26]; (3) and the increase in ROS level by the increase in NOX3 [56] and the increase in intracellular sodium which promote calcium influx via the sodium–calcium exchanger (NCX) [37]. The latter may in part explain the fetal intracellular calcium overload observed during in utero cardiomyocyte development in HCMH. All this should be verified during fetal development of HCMH cardiomyocytes.

Our results suggest that NHE-1 played an important role in heart development, and its increased density during fetal development could be an early marker for hereditary cardiomyopathy. Our results are consistent with the growing evidence for the important role of NHE-1 in the postnatal development of cardiac pathologies associated with intracellular Na^+^ and Ca^2+^ overload, such as hypertrophy and heart failure [37].

Several questions remained: (1) if the blockade of NHE-1 prevents the development of postnatal HCMH and early death [37], does blockade of NHE-1 during fetal development prevent the postnatal and adult development of hereditary cardiomyopathy? (2) Does the blockade of intracellular ROS overload during in utero development of HCMH cardiomyocytes prevent the postnatal development of hereditary cardiomyopathy ? This should be done in the future.

## 5. Conclusions

Our results suggest that hereditary cardiomyopathy took place during fetal development and that the associated remodeling continued throughout life, such as necrosis, hypertrophy, and early death. Targeting the NHE-1 and ROS generation during fetal development of hereditary cardiomyopathy could be an excellent therapeutic means for the prevention of genetic cardiac diseases associated with intracellular Na^+^ overload, such as hypertrophy and premature death in hereditary cardiomyopathy.

## Data Availability

The original contributions presented in this study are included in the article. Further inquiries can be directed to the corresponding authors.
